# Analysis of the Reformulated Source to Drain Tunneling Probability for Improving the Accuracy of a Multisubband Ensemble Monte Carlo Simulator

**DOI:** 10.3390/mi13040533

**Published:** 2022-03-28

**Authors:** Jose Luis Padilla, Cristina Medina-Bailon, Antonio Palomares, Luca Donetti, Carlos Navarro, Carlos Sampedro, Francisco Gamiz

**Affiliations:** 1 Nanoelectronics Research Group, Departamento de Electrónica y Tecnología de Computadores, Universidad de Granada, 18071 Granada, Spain; cmedba@ugr.es (C.M.-B.); donetti@ugr.es (L.D.); carlosnm@ugr.es (C.N.); csampe@ugr.es (C.S.); fgamiz@ugr.es (F.G.); 2 Centro de Investigación en Tecnologías de la Información y las Comunicaciones, Universidad de Granada, 18071 Granada, Spain; 3 Departamento de Matemática Aplicada, Universidad de Granada, 18071 Granada, Spain; anpalom@ugr.es

**Keywords:** direct source-to-drain tunneling, tunneling probability, multi-subband ensemble Monte Carlo

## Abstract

As an attempt to improve the description of the tunneling current that arises in ultrascaled nanoelectronic devices when charge carriers succeed in traversing the potential barrier between source and drain, an alternative and more accurate non-local formulation of the tunneling probability was suggested. This improvement of the probability computation might result of particular interest in the context of Monte Carlo simulations where the utilization of the conventional Wentzel-Kramers-Brillouin (WKB) approximation tends to overestimate the number of particles experiencing this type of direct tunneling. However, in light of the reformulated expression for the tunneling probability, it becomes of paramount importance to assess the type of potentials for which it behaves adequately. We demonstrate that, for ensuring boundedness, the top of the potential barrier cannot feature a plateau, but rather has to behave quadratically as one approaches its maximum. Moreover, we show that monotonicity of the reformulated tunneling probability is not guaranteed by boundedness and requires an additional constraint regarding the derivative of the prefactor that modifies the traditional WKB tunneling probability.

## 1. Introduction

The physical phenomenon of quantum mechanical tunneling between source and drain (S/D tunneling) arises when the scaling process of electronic devices enters the regime of channel lengths around and below 10 nm [1,2]. Monte Carlo simulations aiming to account for the description of this type of structures (when this tunneling contribution requires attention in the subthreshold regime) were traditionally providing current levels above those obtained from simulators with full quantum transport treatment [3]. In spite of this drawback, the design and conception of a Multisubband Ensemble Monte Carlo (MS-EMC) tool still provides a very efficient computation technique based on a modular implementation of the specific quantum transport mechanisms involved in the assessment of each device of interest [4,5]. For that reason, the improvement of the MS-EMC tool in order to achieve a satisfactory description of the S/D tunneling phenomena is particularly valuable in the context of the present sizes of ultrascaled devices.

To do so, one of the proposed solutions turned out to be the reformulation of the tunneling probability across the potential barrier between source and drain [6]. This was implemented by means of a non-local approach following the formalism developed in [7], that introduces an additional term correcting the traditional WKB tunneling probability for 2D simulations. Labeling *x* as the transport direction and *z* the perpendicular direction affected by confinement, the modified tunneling probability reads as [8]

(1) $$T_{\mathrm{DT}}\left(E_{x}\right)=\frac{\Delta_{y}}{2\sqrt{\pi }}{\left[\hbar \int_{a}^{b}\frac{dx}{\sqrt{2m_{x}\left(E_{i}\left(x\right)-E_{x}\right)}}\right]}^{-1/2}\cdot T_{\mathrm{WKB}}\left(E_{x}\right),$$

where $\Delta_{y}$ is the mesh spacing in the periodic direction normal to both transport and confinement dimensions; *a* and *b* are the limits of the tunneling path; $m_{x}$ is the tunneling effective mass of the electron; $E_{x}$ is the total energy of the superparticle in the transport plane considering only the projection of the kinetic energy in the direction facing the barrier, and $E_{i}\left(x\right)$ stands for the energy profile of the *i*-th subband. $T_{\mathrm{WKB}}$ represents the conventional tunneling probability given by the WKB approximation and reads as

(2) $$T_{\mathrm{WKB}}\left(E_{x}\right)=\exp\left[-\frac{2}{\hbar }\int_{a}^{b}\;\sqrt{2m_{x}\left(E_{i}\left(x\right)-E_{x}\right)}\;dx\right].$$

In light of the expression for $T_{\mathrm{DT}}$, its behavior needs to be assessed (due to the term with the negative power) in order to determine for which types of potential barriers it can be used as an appropriate tunneling probability. Should this analysis not be carried out, one might risk to derive untrustworthy conclusions about the S/D importance in those devices where it plays a non-negligible role.

Notice that, in an alternative way to the stochastic conception implied bythe Monte Carlo method, the problem of the lack of validity of the WKB approximation at the turning points of the potential barrier could also be envisaged from a different theoretical perspective employing a wave function formalism. Should this approach be followed, the wave functions would need to be described in in the neighborhoods of the turning points by asymptotic formulas whose leading term would be expressed in terms of Bessel functions.

This paper analyzes the mathematical behavior of the proposed $T_{\mathrm{DT}}$ tunneling probability in the context of the utilizaton of a MS-EMC simulator and delimits the type of potentials for which it proves to be suitable. In Section 2, we expose the behavior of $T_{\mathrm{DT}}$ for a very simple triangular potential profile, illustrating the nature of the problems to be faced. In Section 3, we focus on realistic smooth potentials like those provided by solving the Poisson equation and derive the conditions to be fulfilled by them in order to guarantee an adequate utilization of $T_{\mathrm{DT}}$ when simulating with the MS-EMC tool. Finally, the main conclusions are drawn in Section 4.

## 2. Reformulated Tunneling Probability Applied to a Simple Potential Barrier

When the carrier impinges on the energy barrier with a certain energy $E_{x}$, it is obvious that $E_{i}\left(x\right)$ equals $E_{x}$ at the point where the carrier enters the barrier (i.e., the point with x=a in the integrals above). The problem arises as long as this leads to a divergence in the integrand of the term with power $-\frac{1}{2}$ in $T_{\mathrm{DT}}$ (which is something not to be concerned about if one simply handles with tunneling probabilities described by $T_{\mathrm{WKB}}$).

In what follows, and for the ease of treatment, all auxiliary constants will be set to one and $T_{\mathrm{DT}}$ will be written as

(3) $$T_{\mathrm{DT}}\left(E_{x}\right)={\left[G(E_{x})\right]}^{-\frac{1}{2}}\exp^{-H(E_{x})},$$

with

(4) $$\begin{array}{ccc} G\left(E_{x}\right)=\int_{a(E_{x})}^{b(E_{x})}g\left(E_{x},x\right)\;dx, & \; & H\left(E_{x}\right)=\int_{a(E_{x})}^{b(E_{x})}h\left(E_{x},x\right)\;dx, \end{array}$$

and

(5) $$g\left(E_{x},x\right)=\frac{1}{h(E_{x},x)}=\frac{1}{\sqrt{E_{i}\left(x\right)-E_{x}}}.$$

**Figure 1:**
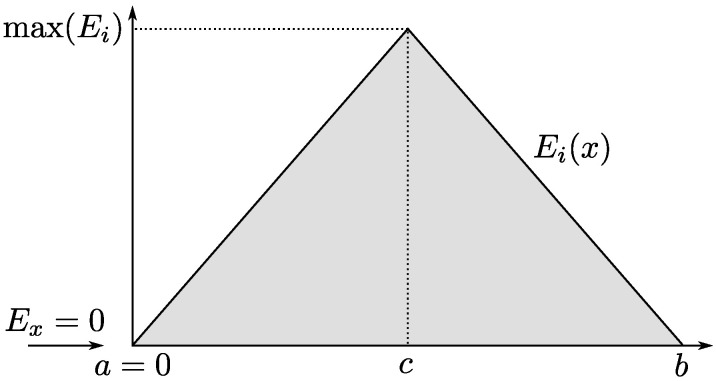
Triangular barrier profile where the carrier impinges on the barrier with $E_{x}=0$. If the carrier suceeds in traversing the barrier, it exits at the point x=b with the same energy.

Let us now consider the elementary triangular barrier depicted in Figure 1 given by

(6) $$\begin{array}{c} E_{i}\left(x\right)=\left\{\begin{array}{ccc} x & \; & a\leq x\leq c\\ \; & \; & \\ -(x-b) & \; & c<x\leq b \end{array}\right.. \end{array}$$

We will suppose that the barrier starts at x=0 (i.e., a=0) and that the carrier hits the barrier with $E_{x}=0$.

In that case, $G(E_{x})$ would read as

(7) $$G\left(E_{x}\right){|}_{E_{x}=0}=\int_{0}^{b}\frac{dx}{\sqrt{E_{i}\left(x\right)}}=2\int_{0}^{c}\frac{dx}{\sqrt{x}}=4{\left[\sqrt{x}\right]}_{0}^{c}=4\sqrt{c}.$$

where the divergence disappears thanks to the integral. However, for this simple example, the resulting behavior of $T_{\mathrm{DT}}$ is incompatible with that corresponding to a well behaved tunneling probability, namely:

(8) $$\begin{array}{c} \lim_{E_{x}\to \max\left(E_{i}\right)}G\left(E_{x}\right)=0\Rightarrow \lim_{E_{x}\to \max\left(E_{i}\right)}T_{\mathrm{DT}}\left(E_{x}\right)=\infty \end{array}$$

Therefore, careful assessment needs to be carried out as to determine for which type of potential barriers the utilization of $T_{\mathrm{DT}}$ might be suitable.

## 3. Reformulated Tunneling Probability Applied to Smooth Potential Barriers

Let us now consider the situation where the potential barrier can be described by a smooth differentiable function between the limits of integration. This situation differs from the simplified scenario analyzed in the previous section (as far as the triangular barrier was not smooth at its maximum), but proves to be more realistic taking into account the usual shape of the potential barriers to be found in scenarios where source to drain tunneling arises.

### 3.1. Boundedness

According to the form of $g(E_{x},x)$, and in order to keep its integral finite when evaluating it at x=a for a given $E_{x}<\max\left(E_{i}\right)$, we need that

(9) $$\lim_{x\to a(E_{x})}g\left(Ex,x\right)<\lim_{x\to a(E_{x})}\frac{1}{x-a(E_{x})}.$$

Or, in other words, that

(10) $$\lim_{x\to a(E_{x})}\left[x-a(E_{x})\right]g\left(E_{x},x\right)=\lim_{x\to a(E_{x})}\frac{x-a(E_{x})}{\sqrt{E_{i}\left(x\right)-E_{x}}}=0.$$

If we compute this limit for an energy barrier $E_{i}\left(x\right)$, we see that both numerator and denominator tend to zero as *x* approaches $a(E_{x})$. For that reason, the limit can be computed by l’Hôpital as

(11) $$\lim_{x\to a(E_{x})}\frac{x-a(E_{x})}{\sqrt{E_{i}\left(x\right)-E_{x}}}=\lim_{x\to a(E_{x})}\frac{1}{\frac{1}{2\sqrt{E_{i}\left(x\right)-E_{x}}}E_{i}^{\prime }\left(x\right)}=\lim_{x\to a(E_{x})}\frac{2\sqrt{E_{i}\left(x\right)-E_{x}}}{E_{i}^{\prime }\left(x\right)},$$

that equals zero provided that the barrier has a certain slope at the impinging point $a(E_{x})$, which is inherent to the nature of the barrier itself. The reasoning is exactly analogous for the exit point $b(E_{x})$. Observe that this result is independent of the shape of $E_{i}\left(x\right)$, the only requirement is that $E_{i}^{\prime }\neq 0$ at $a(E_{x})$ and $b(E_{x})$.

Up to this point, the only thing that is guaranteed is that $G(E_{x})$ will remain finite for $E_{x}<\max\left(E_{i}\right)$ provided that $E_{i}^{\prime }\neq 0$ at $a(E_{x})$ and $b(E_{x})$. However, we still need $G(E_{x})$ not to go to zero or to infinity as $E_{x}$ tends to $\max(E_{i})$. As we are supposing $E_{i}\left(x\right)$ to be smooth, it can be expanded in Taylor series around the point $x_{0}$ where $E_{i}\left(x\right)$ attains its maximum. This leads to

(12) $$E_{i}\left(x\right)=c_{0}+c_{1}\left(x-x_{0}\right)+c_{2}{\left(x-x_{0}\right)}^{2}+c_{3}{\left(x-x_{0}\right)}^{3}+c_{4}{\left(x-x_{0}\right)}^{4}+\ldots ,$$

with $c_{0}=\max\left(E_{i}\right)$, $c_{1}=0$ and $c_{2}\leq 0$. Now suppose that, for simplicity, the potential is centered at the origin so that $x_{0}=0$. The above expression reduces to

(13) $$E_{i}\left(x\right)=\max\left(E_{i}\right)+c_{2}\;x^{2}+c_{3}\;x^{3}+c_{4}\;x^{4}+\ldots$$

Since we are interested in the behavior when $E_{x}$ tends to $\max(E_{i})$, this is the same as saying that we are interested in the behavior of the Taylor series when *x* tends to zero or, in other words, that the key of the analysis relies on the order of the first non vanishing coeficient of the expansion, which is the one controlling how $E_{i}\left(x\right)$ approaches to its maximum.

Let us suppose that $c_{2}\neq 0$ (which leads to $c_{2}<0$ because the potential has a maximum). In that case, we can neglect all the terms of higher order and obtain a simple inverted parabolic potential. If we compute the value of $G(E_{x})$, we obtain

(14) $$\begin{array}{cc} G(E_{x}) & =\int_{-\frac{\sqrt{\max\left(E_{i}\right)-E_{x}}}{\sqrt{-c_{2}}}}^{\frac{\sqrt{\max\left(E_{i}\right)-E_{x}}}{\sqrt{-c_{2}}}}\frac{dx}{\sqrt{\max\left(E_{i}\right)+c_{2}x^{2}-E_{x}}}\\ \; & =\frac{1}{\sqrt{-c_{2}}}{\left[\arcsin\left(\frac{\sqrt{-c_{2}}}{\sqrt{\max\left(E_{i}\right)-E_{x}}}\;x\right)\right]}_{-\frac{\sqrt{\max\left(E_{i}\right)-E_{x}}}{\sqrt{-c_{2}}}}^{\frac{\sqrt{\max\left(E_{i}\right)-E_{x}}}{\sqrt{-c_{2}}}}\\ \; & =\frac{2}{\sqrt{-c_{2}}}\arcsin\left(1\right)=\frac{\pi }{\sqrt{-c_{2}}}. \end{array}$$

This expression is independent of $E_{x}$ and remains constant when $E_{x}$ tends to $\max(E_{i})$, thus providing a finite non zero value of $G(E_{x})$ even at the top of the barrier.

On the other hand, if $c_{2}=0$ (which means that the barrier will feature a certain plateau at its top), the next non vanishing (and negative) coeficient will necessarily be $c_{j}$ with *j* even, and again all orders above it can be neglected if we want to analyze the behavior when $E_{x}$ tends to $\max(E_{i})$. The corresponding expression for $G(E_{x})$ is then

(15) $$\begin{array}{cc} G(E_{x}) & =\int_{a(E_{x})}^{b(E_{x})}\frac{dx}{\sqrt{\max\left(E_{i}\right)+c_{j}x^{j}-E_{x}}}\;\left(c_{j}<0,\;j=4,6,8,\ldots \right), \end{array}$$

with

(16) $$\begin{array}{ccc} a\left(E_{x}\right)=-{\left(\frac{\max\left(E_{i}\right)-E_{x}}{-c_{j}}\right)}^{\frac{1}{j}}, & \; & b\left(E_{x}\right)={\left(\frac{\max\left(E_{i}\right)-E_{x}}{-c_{j}}\right)}^{\frac{1}{j}}. \end{array}$$

The computation of $G(E_{x})$ by means of elliptic integrals leads to

(17) $$G\left(E_{x}\right)=\frac{2\sqrt{\pi }\;\Gamma \left(\frac{j+1}{j}\right)}{{\left(-c_{j}\right)}^{\frac{1}{j}}{\left[\left(\max\left(E_{i}\right)-E_{x}\right)\right]}^{\frac{\frac{j}{2}-1}{j}}\;\Gamma (\frac{\frac{j}{2}+1}{j})}\;\left(c_{j}<0,\;j=4,6,8,\ldots \right),$$

where Γ stands for the gamma function. The presence of the term $\left(\max\left(E_{i}\right)-E_{x}\right)$ in the denominator makes $G(E_{x})$ diverge as $E_{x}$ approaches $\max(E_{i})$. This result indicates that the potential barrier must necessarily possess a cuadratic behavior close to its maximum, and that the appearance of an upper plateau (for example, if the gate length is not too aggressively scaled) deprives $T_{\mathrm{DT}}$ from its physical meaning. Fortunately, given that S/D tunneling becomes relevant for narrow potentials, this requirement will very likely be satisfied in most of the cases of interest.

### 3.2. Monotonicity

The fact of assuring that $G(E_{x})$ remains bounded and non zero for any of the impinging energies of interest is not enough for guaranteeing an appropriate behavior of $T_{\mathrm{DT}}$. We need to demand monotonicity too. In other words, the tunneling probability needs to increase as $E_{x}$ approaches $\max(E_{i})$.

**Figure 2:**
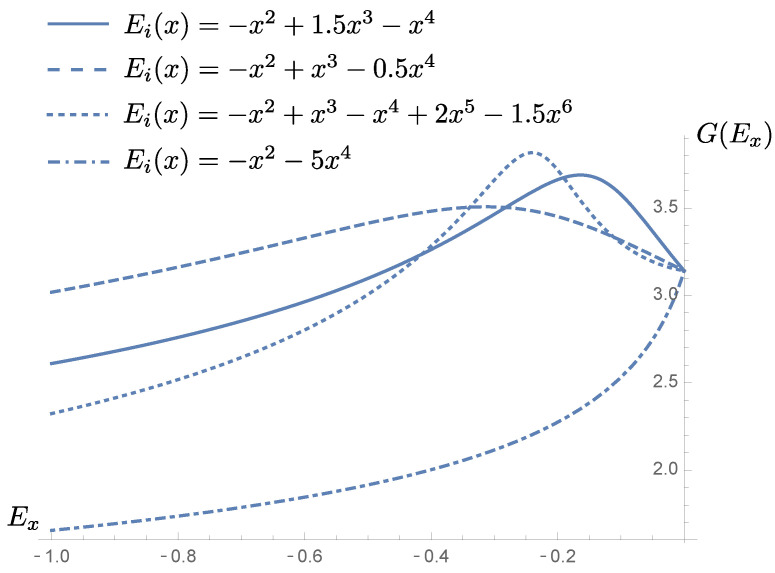
Behavior of the factor $G(E_{x})$ for different potential barriers $E_{i}\left(x\right)$ with $\max(E_{i})=0$. Notice how, as $E_{x}\to \max\left(E_{i}\right)$, $G(E_{x})$ tends to the finite expected value of $\frac{\pi }{\sqrt{-c_{2}}}=\pi$.

Note that, in principle, and from the form of $T_{\mathrm{DT}}$ in Equation (3), one cannot affirm that this will always be the case since $T_{\mathrm{DT}}$ results from the product of a function of $G(E_{x})$ and a function of $H(E_{x})$, and $G(E_{x})$ can very well not be monotonic as shown in Figure 2 for different barrier examples fulfilling boundedness. It is therefore desirable to derive a condition that ensures the monotonicity of $T_{\mathrm{DT}}$ once the shape of the potential is known.

For that aim, we shall demand that $T_{\mathrm{DT}}^{\prime }\left(E_{x}\right)\geq 0$. In our case, and considering that $T_{\mathrm{DT}}$ always evaluates to a positive number, we can use that

(18) $$T_{\mathrm{DT}}^{\prime }\left(E_{x}\right)\geq 0\Leftrightarrow {\left[\ln T_{\mathrm{DT}}\left(E_{x}\right)\right]}^{\prime }\geq 0,$$

which proves to be a more convenient condition for studying the behavior of $T_{\mathrm{DT}}$ recalling its expression as a function of $G(E_{x})$ and $H(E_{x})$. Therefore, using Equation (3) we obtain that

(19) $${\left[\ln T_{\mathrm{DT}}\left(E_{x}\right)\right]}^{\prime }\geq 0\Leftrightarrow -\frac{1}{2}\frac{G^{\prime }\left(E_{x}\right)}{G(E_{x})}-H^{\prime }\left(E_{x}\right)\geq 0\Leftrightarrow \frac{1}{2}\frac{G^{\prime }\left(E_{x}\right)}{G(E_{x})}+H^{\prime }\left(E_{x}\right)\leq 0.$$

For calculating the derivatives of $G(E_{x})$ and $H(E_{x})$ we need to apply the Leibniz rule, which leads to

(20) $$\begin{array}{c} G^{\prime }\left(E_{x}\right)=\int_{a(E_{x})}^{b(E_{x})}\frac{\partial g(E_{x},x)}{\partial E_{x}}\;dx+g\left(E_{x},b\left(E_{x}\right)\right)b^{\prime }\left(E_{x}\right)-g\left(E_{x},a\left(E_{x}\right)\right)a^{\prime }\left(E_{x}\right), \end{array}$$

(21) $$\begin{array}{c} H^{\prime }\left(E_{x}\right)=\int_{a(E_{x})}^{b(E_{x})}\frac{\partial h(E_{x},x)}{\partial E_{x}}\;dx+h\left(E_{x},b\left(E_{x}\right)\right)b^{\prime }\left(E_{x}\right)-h\left(E_{x},a\left(E_{x}\right)\right)a^{\prime }\left(E_{x}\right). \end{array}$$

Taking the relationship between $g(E_{x},x)$ and $h(E_{x},x)$ of Equation (5), we observe that

(22) $$\begin{array}{cc} \frac{\partial h(E_{x},x)}{\partial E_{x}} & =-\frac{1}{2\sqrt{E_{i}\left(x\right)-E_{x}}}=-\frac{1}{2}g\left(E_{x},x\right), \end{array}$$

(23) $$\begin{array}{cc} \frac{\partial g(E_{x},x)}{\partial E_{x}} & =\frac{\partial }{\partial E_{x}}\left(\frac{1}{h(E_{x},x)}\right)=-\frac{1}{h^{2}\left(E_{x},x\right)}\;\frac{\partial h(E_{x},x)}{\partial E_{x}}=\frac{1}{2}g^{3}\left(E_{x},x\right). \end{array}$$

Which provides

(24) $$\begin{array}{cc} G^{\prime }\left(E_{x}\right) & =\frac{1}{2}\int_{a(E_{x})}^{b(E_{x})}g^{3}\left(E_{x},x\right)\;dx+g\left(E_{x},b\left(E_{x}\right)\right)b^{\prime }\left(E_{x}\right)-g\left(E_{x},a\left(E_{x}\right)\right)a^{\prime }\left(E_{x}\right), \end{array}$$

(25) $$\begin{array}{cc} H^{\prime }\left(E_{x}\right) & =-\frac{1}{2}G\left(E_{x},x\right)+h\left(E_{x},b\left(E_{x}\right)\right)b^{\prime }\left(E_{x}\right)-h\left(E_{x},a\left(E_{x}\right)\right)a^{\prime }\left(E_{x}\right). \end{array}$$

Now, since $h(E_{x},x)$ evaluates to zero when $x=a(E_{x})$ and $x=b(E_{x})$, the last equation is simply

(26) $$H^{\prime }\left(E_{x}\right)=-\frac{1}{2}G\left(E_{x}\right).$$

If we apply this result to Equation (19) and use that $G(E_{x})$ is always positive, we obtain that

(27) $$\frac{1}{2}\frac{G^{\prime }\left(E_{x}\right)}{G(E_{x})}+H^{\prime }\left(E_{x}\right)\leq 0\Leftrightarrow G^{\prime }\left(E_{x}\right)\leq G^{2}\left(E_{x}\right)\Leftrightarrow {\left(\frac{1}{G(E_{x})}\right)}^{\prime }\geq -1.$$

**Figure 3:**
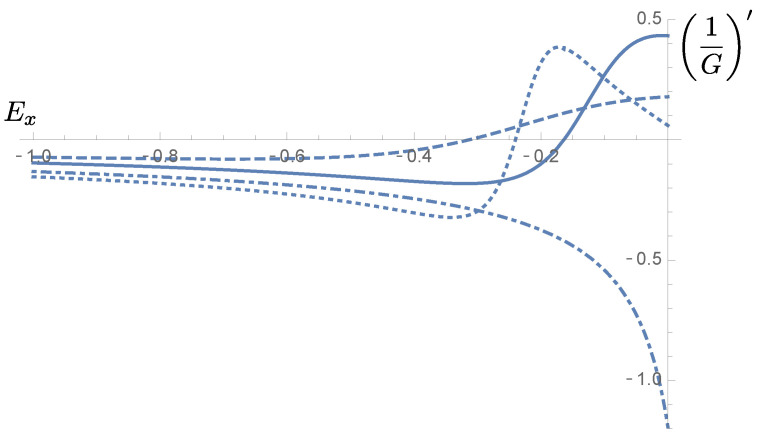
Behavior of the derivative of $1/G(E_{x})$ for the potential barriers considered in Figure 2. Three of them fulfill the condition that ensures monotonicity for $T_{\mathrm{DT}}$, whereas one does not.

This last inequality constitutes a simple way of formulating the condition that must be satisfied once the shape of the potential barrier $E_{i}\left(x\right)$ is known in order to guarantee the monotonicity of $T_{\mathrm{DT}}$. In Figure 3, we observe that three of the example barriers of Figure 2 fulfill this condition, so that the probability $T_{\mathrm{DT}}$ would be well behaved for them. On the other hand, for the dashed dotted curve, the probability $T_{\mathrm{DT}}$ would not be monotonic.

We conclude that boundedness does not necessarily imply monotonicity for $T_{\mathrm{DT}}$. For a given potential, both requirements would need to be verified separately.

## 4. Conclusions

One of the envisageable solutions to improve the accuracy of the S/D tunneling current computation in a MS-EMC simulator is to redefine the tunneling probability by which it is estimated. However, in light of the resulting expression obtained from a non-local reformulation, its applicability is not automatically well defined for any potential barrier that might arise between source and drain regions. We have demonstrated that boundedness of the tunneling probability is ensured for potentials not featuring a flat region around its maximum (i.e., those with non-vanishing quadratic terms when expanded in series). With respect to monotonicity, this property is not automatically ensued from the boundedness of the tunneling probability and requires, in turn, a certain constraint on the boundedness of the derivative of the prefactor modifying the WKB tunneling probability.

## Data Availability

Not applicable.

## Abbreviations

The following abbreviations are used in this manuscript:

S/D	Source to drain
MS-EMC	Multisubband Ensemble Monte Carlo
WKB	Wentzel-Kramers-Brillouin
